# Genome-Wide Identification and Tissue-Specific Expression Profiling of Goji *CER* Gene Family

**DOI:** 10.3390/genes16111257

**Published:** 2025-10-24

**Authors:** Qian Yu, Jie Li, Lijuan Jing, Feng Zhang, Bohua Liu, Liuwei Guo

**Affiliations:** 1 Gansu Analysis and Research Center, Lanzhou 730000, China; gfcyq09@163.com (Q.Y.); zfgood_303@163.com (F.Z.); 2 College of Forestry, Gansu Agricultural University, Lanzhou 730070, China; jlj0529@sina.com (L.J.); 18793961096@163.com (L.G.); 3 Wolfberry Harmless Cultivation Engineering Research Center of Gansu Province, Lanzhou 730070, China; 4 College of Horticulture, Gansu Agricultural University, Lanzhou 730070, China; 1073323120849@st.gsau.edu.cn

**Keywords:** goji cuticular wax, *CER* gene family, expression analysis, cultivar, organs

## Abstract

Background: Goji berry, known as a “superfood”, is widely distributed in northwest China and possesses significant medicinal and health value. The *CER* gene family serves as a key regulator of cuticular wax synthesis, which plays important roles in enhancing plant drought resistance and disease tolerance. However, genome-wide identification of the goji *CER* gene family and its expression analysis across different varieties and organs have not been reported. Methods: Based on SEM observations and wax load measurements, this study identified *CER* gene family members using whole genome data of the goji berry. Representative genes were selected and their expression patterns in different varieties and organs were validated by qRT–PCR. Results: The stem wax load was significantly higher than that in other organs, while the leaf wax load of ‘Ningqi I’ goji was significantly higher than that in other varieties, consistent with SEM observations. A total of 113 *CER* gene family members were identified in goji berry, which were unevenly distributed on 12 chromosomes. The goji CER proteins mainly localized in the cell membrane, cytoplasm, chloroplast, and nucleus and clustered into five subfamilies. Ten conserved motifs were identified in CER proteins, with Motif5 and Motif7 being the most widely distributed. The *LbaCER10-1* gene contained the highest number of exons (39). Cis-acting elements related to light-responsiveness, MeJA-responsiveness, and ABA-responsiveness showed high frequencies. Goji berry shared more homologous *CER* genes with tomato, potato, and tobacco than with Arabidopsis, with chr3 and chr9 being most conserved while chr7 showed greater variation. Conclusions: Integrating SEM, wax load, and qRT–PCR results, *LbaCER1-1* was identified as a candidate gene responsible for the higher wax load on goji stems, while *LbaCER2-5* and *LbaCER3-12* were candidate genes for greater wax load on ‘Ningqi I’ leaves.

## 1. Introduction

Goji berry (*Lycium barbarum*), a perennial shrub species belonging to the Solanaceae family, exhibits remarkable cold resistance [1], drought tolerance [2], and saline–alkali endurance [3], endowing it with significant ecological importance. As a predominant economic crop cultivated in Northwest China, this species possesses substantial economic and medicinal value [4]. Globally recognized by consumers, it has been widely acclaimed as a “superfood” [5,6].

The plant cuticular wax is a hydrophobic layer covering the epidermal cells of all terrestrial plant aerial parts, serving as a crucial physical barrier that plants have developed to adapt to terrestrial environments [7]. It plays important roles in protecting against UV damage [8,9], maintaining plant surface cleanliness [10], resisting pathogen and pest attacks [11,12], and preventing non-stomatal water loss [13,14]. Plant cuticular wax is primarily composed of very-long-chain fatty acids (VLCFAs) and their derivatives, including both aliphatic compounds (alkanes, alkenes, aldehydes, alcohols, ketones, and terpenoids) and aromatic compounds (phenylpropanoids, polyphenols, and flavonoids) [15,16]. The eceriferum (*CER*) gene family is one of the major gene families regulating cuticular wax biosynthesis. To date, *CER* gene family members have been reported in several plant species, including *Arabidopsis thaliana* [17], maize (*Zea mays*) [18], cucumber (*Cucumis sativus*) [19], wheat (*Triticum aestivum*) [20], tomato (*Solanum lycopersicum*) [21], sunflower (*Helianthus annuus*) [22], jujube (*Ziziphus jujuba*) [23], chestnut (*Castanea mollissima*) [24], barley (*Hordeum vulgare*) [25], and pepper (*Capsicum annuum*) [26]. Extensive functional studies have been conducted on the *CER* gene family in *Arabidopsis*. The *AtCER1* and *AtCER3* complexes serve as key enzymes in the alkane biosynthesis pathway, jointly catalyzing the formation of alkanes from VLCFA-CoA [27]. *AtCER2* is a regulatory protein of the fatty acid elongase complex, acting on C28 elongation and showing epidermis-specific expression [28,29]. Inactivation of *AtCER16* suppresses *AtCER3* expression, thereby affecting alkane production [30]; *AtWBC11* interacts with *AtCER5* to facilitate the secretion of surface waxes [31]. Other members studied include *AtCER6* [32], *AtCER7* [33,34], *AtCER8* [35], *AtCER10* [36], *AtCER11* [37], and *AtCER17* [38]. In other plant species, functional studies have revealed diverse roles for *CER* genes. For example, *CsCER1* in cucumber influences VLC alkane biosynthesis and drought resistance [39]. The apple (*Malus pumila*) *MdCER* gene family responds to PEG-induced drought stress [40]. In tomato, the multicellular trichome regulator *Woolly* and *SlMYB31* coordinately regulate *SlCER6* expression to control cuticular wax biosynthesis [21]. The *SlCER1-1* gene can regulate n-alkane and branched alkane content and enhance adaptation to drought stress [41]. In *Populus* × *canescens*, *CER6* gene mutation leads to significant changes in wax composition but not in total wax quantity [42].

The functional roles of goji cuticular wax have been investigated in several aspects including drought resistance in leaves [43], powdery mildew resistance [44], and postharvest fruit storage [45]. Several functional genes, such as *CER1*, *CER6*, *FAR*, *MAH1*, and *MYB96*, have been identified. However, fundamental information regarding the entire goji *CER* gene family remains unclear, particularly concerning the number of family members, their genomic localization, structural characteristics, and expression patterns across different cultivars and organs. In this study, we utilized whole-genome sequencing data of goji berry and employed *Arabidopsis* *CER* gene family protein structures as reference models to identify all *CER* gene family members. Through comprehensive bioinformatics analyses, we characterized various features of these family members, including protein physicochemical properties, chromosomal localization, synteny relationships, conserved motifs, cis-acting elements, and evolutionary relationships. Finally, by integrating domain architecture analysis with transcriptome data, we selected five representative genes for expression profiling across different cultivars and organs. These expression patterns were correlated with wax load measurements and SEM observations, aiming to elucidate the relationship between goji *CER* gene family expression and cuticular wax deposition. This research provides theoretical support for multiple applications, including goji berry processing and storage, exploration of drought resistance mechanisms, and breeding of disease-resistant cultivars.

## 2. Materials and Methods

### 2.1. Plant Materials

The plant materials were obtained from ‘Ningqi I’, ‘Ningnongqi XVI’, and ‘Huangguo’ goji, three cultivars planted in spring 2022 in the field of the Economic Forest Training Base of the College of Forestry, Gansu Agricultural University, located in Anning District, Lanzhou City, Gansu Province. All three cultivars received identical cultivation management, including fertilization, irrigation, pruning, and pest control, during their growth period. In mid-July 2025, samples of stems, leaves, flowers, and fruits were collected from each cultivar for scanning electron microscopy (SEM) observation, cuticular wax load measurement, and quantitative real-time PCR (qRT–PCR) analysis. Three individual plant samples from each variety were collected. For qRT–PCR analysis, the collected materials were immediately frozen in liquid nitrogen and stored at −80 °C until use.

### 2.2. The Cuticular Wax Crystallization Patterns

SEM imaged the cuticular wax crystallization patterns. The plant materials were stored overnight in a 5% glutaraldehyde fixation solution at 4 °C and then transferred to a supercritical dryer to dehydrate for more than 4 h [46]. The dried samples were coated with gold for 2 min in a sputter coater (MC1000, Hitachi, Tokyo, Japan) and observed via SEM (S3400N, Hitachi, Tokyo, Japan). The plant materials were placed facing up and subjected to SEM, at an accelerating voltage of 5 kV; afterwards, the leaves were observed, and photos were taken with six replicates.

### 2.3. The Cuticular Wax Load Analysis

The wax load of plant materials was determined by the chloroform method [47]. Firstly, the wax-extracted materials were scanned using a scanner (Perfection V700 Photo, Epson, Suwa, Japan), and the area was calculated using Image J software (version 1.54). The materials were eluted with chloroform for 45 s. The chloroform had to completely submerge the extraction materials. We weighed the empty beaker and the beaker after extraction and drying to constant weight. The difference was the weight of the wax. The wax load was expressed as μg/cm^2^. Each sample analysis was repeated three times.

### 2.4. Identification and Physicochemical Characterization of CER Gene Family Members

The annotated *CER* sequences of *A. thaliana* were downloaded from the *Arabidopsis* genome annotation website (https://www.arabidopsis.org/, accessed on 9 August 2025). The goji plants’ genome data were downloaded from the goji (*L. barbarum*) genome database (https://figshare.com/articles/dataset/Goji_genomes_and_the_evolution_of_Lycium_Solanaceae_/20416593, accessed on 6 April 2023) and blasted using BioEdit software (version 7.7), with an E-value threshold of 0.001 for the candidate gene family. Subsequently, using the protein domain architecture of *Arabidopsis* *CER* gene family members as reference, all preliminarily identified goji *CER* genes were methodically validated through the SMART database (https://www.embl.org/sites/heidelberg/, accessed on 10 August 2025) for structural confirmation. Sequences failing to meet the canonical CER protein domain specifications were eliminated.

Online analysis software, ExPASy (https://web.expasy.org/protparam/, accessed on 11 August 2025, version 3.0), was used for the analysis of *CER* genes, encoding amino acid numbers, molecular weight, isoelectric points, and other physical and chemical properties. Then, the protein subcellular localization prediction software Wolfpsort (https://www.genscript.com/, accessed on 11 August 2025) was used for the *LbaCER* family protein sequence analysis to predict subcellular localization.

### 2.5. Chromosome Localization and Synteny Relationship Analysis

The chromosomal localization information of goji *CER* gene family members was extracted from the goji genome annotation file. Chromosomal distribution mapping of goji *CER* genes was subsequently performed using the MG2C online tool (http://mg2c.iask.in/mg2c_v2.1/, accessed on 11 August 2025) [48]. Synteny relationships, including both intraspecies and interspecies analyses, were investigated using TBtools software (version 2.056). The data for the tomato, potato, and tobacco genomes were downloaded from the Sol genomics network (https://solgenomics.net/, accessed on 8 August 2025).

### 2.6. Conserved Domain and Motif Analysis

The goji CER gene protein sequences were submitted to the NCBI-CDD database website (https://www.ncbi.nlm.nih.gov/Structure/cdd/wrpsb.cgi, accessed on 11 August 2025) to obtain the results file. The MEME (https://web.mit.edu/meme/current/share/doc/overview.html, accessed on 11 August 2025, version 4.11.4) website was used for the analysis of goji *CER* genes encoding protein sequences with conservative base sequence analysis. The number of motifs was set to 15. The conserved domain and motifs of goji *CER* genes were visualized using the TBtools (v2.056) software gene structure view (advanced) program.

### 2.7. Cis-Acting Element Analysis

To analyze the cis-acting elements contained in the promoter region, the upstream 2000 bp sequences of 113 goji *CER* genes were extracted using TBtools (v2.056). The online software PlantCARE version 2.0 (http://bioinformatics.psb.ugent.be/webtools/plantcare/html/, accessed on 12 August 2025) was used to predict the cis function components. The TBtools (v2.056) software gene structure view (advanced) was used for visualization.

### 2.8. Phylogenetic Analysis

Multiple sequence alignments of *CER* amino acid sequences for *A. thaliana* and *L. barbarum* were performed using DNAMAN software (version 9.0), and the repeated sequences were removed. Then, MEGA11.0 software was used to determine the optimal conformational tree model of the *CER* gene family for *A. thaliana* and *L. barbarum*. Bootstrap = 1000 to construct the evolutionary tree. The iTOL online website (http://www.evolgenius.info/evolview, accessed on 12 August 2025) was used to beautify the evolutionary tree.

### 2.9. qRT–PCR Assay

RNA was extracted with an RNA Extraction Kit and then diluted to 1000 ng/μL after reverse transcription to obtain cDNA. Primers were designed in the conserved region of the gene by using Primer 5.0 and Oligo 7.0 software and were synthesized by Shanghai Bioengineering Technology Service, Shanghai, China. The gene information and primers used are shown in Table 1. The *RH37* gene was used as a reference gene. The two-step reaction method was used to perform qRT–PCR with a SYBR Green Pro Taq HS qPCR Kit. The reaction system was prepared on ice with 20 μL of 2×SYBR Green Pro Taq HS Premix (10.0 μL), 1 μL each of the upstream and downstream primers (0.4 μmol/mL), 2 μL of cDNA (1000 ng/μL), and 7.2 μL of ddH_2_O. The reaction conditions were as follows: 95 °C predenaturation for 30 s, 95 °C denaturation for 5 s, based on the Tm value of the primer, and 60 °C annealing for 30 s, with a total of 40 cycles. A Light Cycler 96 SW 1.1 (Roche, Basel, Switzerland) was used for qRT–PCR. The number of repeats was *n* = 4. The reaction specificity was determined by analyzing the melting curve, and the cycle threshold (CQ) value of each sample was obtained. The relative expression level of the target gene was calculated using the 2^−ΔΔCt^ method [49,50].

### 2.10. Data Processing

Statistical analysis was performed using GraphPad Prism software (version 10.1.2). One-way analysis of variance (ANOVA) followed by Tukey’s multiple-comparisons test was conducted to determine significant differences among groups. Data are presented as the mean ± SD, with statistical significance defined at *p* < 0.05. Significant differences (*p* < 0.05) are indicated by distinct lowercase letters above each corresponding column in the bar charts.

## 3. Results

### 3.1. Differences in Wax Ultrastructure and Load Across Organs of Different Goji Berry Cultivars

**Figure 1:**
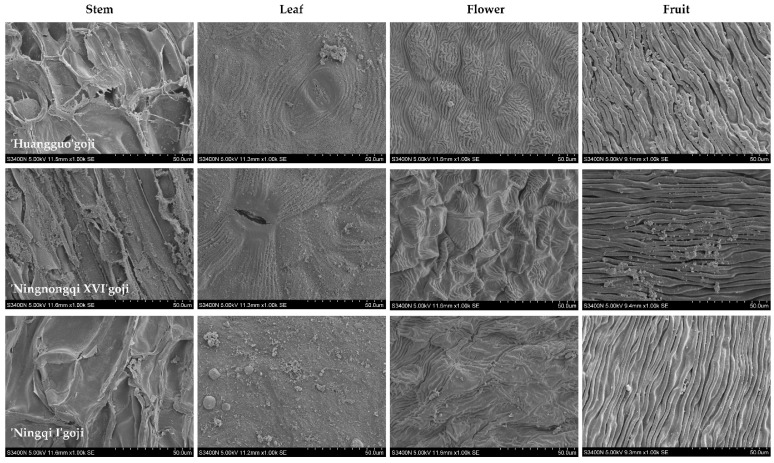
Scanning electron micrographs of the epicuticular wax on the above-ground organs of three goji varieties.

Consistent with previous findings, substantial interspecific variations in both wax load and crystal morphology were documented among the examined goji cultivars. Consequently, we conducted SEM analysis of aerial organs (including stems, leaves, flowers, and fruits) from three representative cultivars, ‘Ningqi I’, ‘Ningnongqi XVI’, and ‘Huangguo’ goji. As shown in Figure 1, the SEM analysis revealed that wax crystals formed dense sheet-like structures on stems, while appearing as granular deposits on leaves, flowers, and fruits across all cultivars. Notably, the wax crystal density on leaf and petal surfaces of ‘Ningqi I’ was significantly higher compared to that of the other two cultivars. No obvious differences in wax ultrastructure were observed among the three cultivars in either stems or fruits.

**Figure 2:**
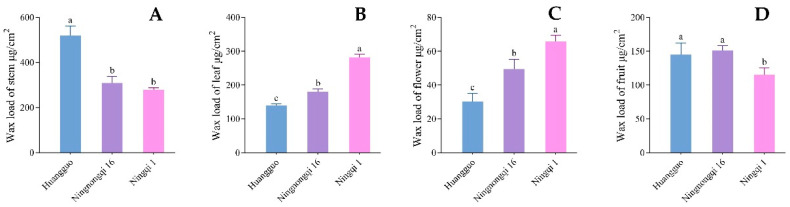
Wax load on above-ground organs of three goji varieties. Note: (**A**) is the wax load of stems; (**B**) is the wax load of leaves; (**C**) is the wax load of flowers; (**D**) is the wax load of young fruit. The letters above bars represents the differences between data. The same letters indicate non-significant differences, while different letters indicate significant differences.

Quantitative analysis of cuticular wax load revealed substantial differences among different cultivars and organs (Figure 2). The stems exhibited the highest wax load (ranging from 279.12 to 520.22 μg/cm^2^), while the flowers showed the lowest wax load (ranging from 30.38 to 65.76 μg/cm^2^). The fruits displayed intermediate wax load (ranging from 115.35 to 151.35 μg/cm^2^), which remained relatively stable. The ‘Huangguo’ goji stems contained 520.22 μg/cm^2^ of wax, significantly greater than that of other cultivars. Notably, the wax load on ‘Ningqi I’ leaves reached 281.95 μg/cm^2^, significantly exceeding that of other cultivars, being 2.02 times greater than that of ‘Huangguo’ goji leaves and 1.56 times greater than that of ‘Ningnongqi XVI’ leaves. Moreover, ‘Ningqi I’ was the only cultivar where leaf wax load surpassed stem wax. The wax load on ‘Ningqi I’ flowers was also significantly greater than that of other cultivars, while its fruit wax content was significantly lower than that of others. These results were largely consistent with observations from SEM.

### 3.2. Identification and Physicochemical Properties of the Goji CER Gene Family Members

To more accurately screen and investigate the possible reasons for the differences in wax load on the goji, we conducted bioinformatics analysis of the *CER* gene family members.

Through HMMER searches and comparison with the *Arabidopsis CER* gene family, a total of 113 *CER* genes were identified in the goji genome. As shown in Supplementary Table S1, the physicochemical properties of the CER proteins in the goji exhibited notable variation, with the number of amino acids ranging from 127 to 2829, molecular weight between 14,413.92 and 320,972.08 kDa, theoretical pI from 4.62 to 9.66, an instability index between 22.69 and 56.53, an aliphatic index from 75.43 to 113.69, and a grand average of hydropathicity (GRAVY) ranging from −0.528 to 0.364. Subcellular localization prediction of the 113 CER proteins revealed their predominant localization in the plasma membrane, cytoplasm, and chloroplasts, among nine organelles. Of these, 101 CER proteins were localized to a single organelle, while 12 proteins exhibited dual localization. Specifically, 54 CER proteins were localized to the plasma membrane, 29 to the cytoplasm, 18 to chloroplasts, 9 to the nucleus, and 7 to peroxisomes. These results indicate that the amino acid length and relative molecular weight of the goji CER proteins exhibit significant variation, and their subcellular localization is highly diverse across different organelles.

### 3.3. Chromosomal Localization and Intra-Genomic Synteny Analysis of the Goji CER Gene Family Members

**Figure 3:**
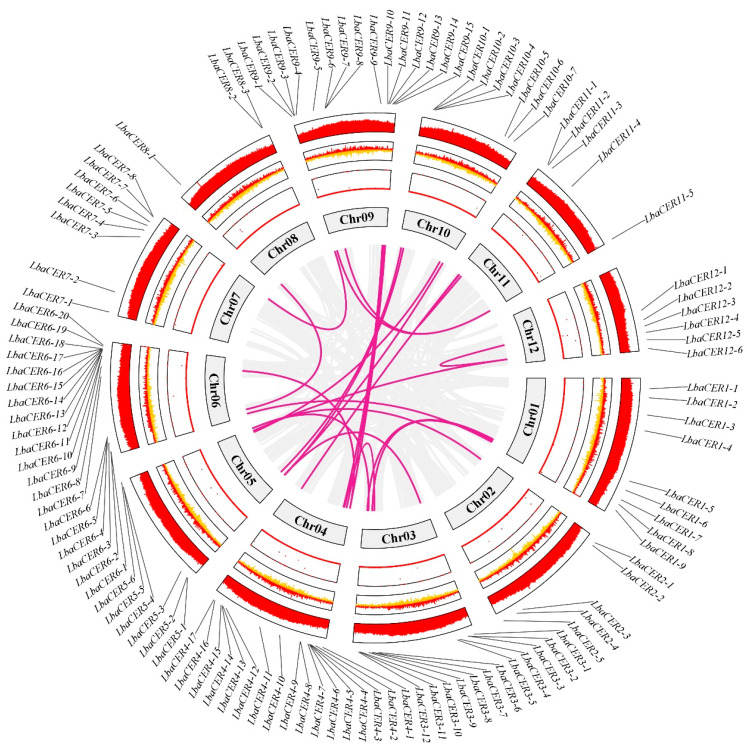
Chromosomal localization and intra-group collinearity analysis of goji *CER* gene family members.

The chromosomal localization of the goji *CER* gene family members is shown in Figure 3. The 113 genes were unevenly distributed across 12 pairs of chromosomes. Among them, chr6 contained the highest number (20 genes), followed by chr4 (17 genes), while chr8 had the fewest (only 3 genes). Most *CER* genes in the goji were arranged in clusters, displaying tandem duplication patterns, with the majority located near the 5′ or 3′ regions.

To investigate gene duplication events, intragenomic synteny analysis of the *CER* gene family was performed. The results revealed 23 syntenic gene pairs within the goji *CER* gene family. Specifically, chr6, chr7, chr11, and chr12 had one syntenic gene pair; chr2, chr3, and chr9 had two syntenic gene pairs; chr1 had three syntenic gene pairs; chr4 and chr5 had five syntenic gene pairs; and chr8 and chr10 had no syntenic gene pairs (Figure 3).

### 3.4. Protein Conserved Motifs and Domain Analysis of Goji CER Gene Family Members

**Figure 4:**
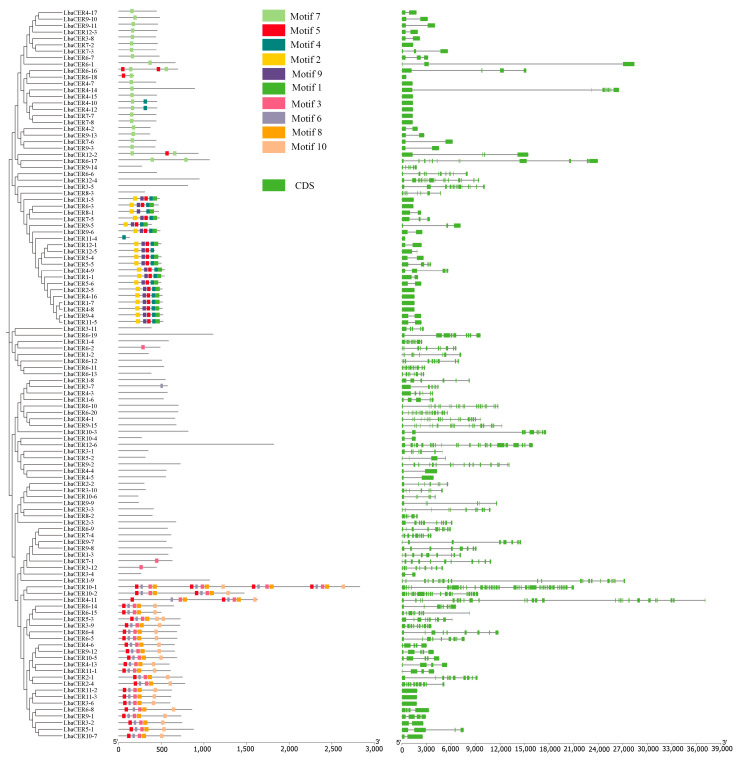
Analysis of conserved motifs and domains in goji *CER* gene family members.

As shown in Figure 4, 10 conserved motifs were identified in goji CER proteins. Among these, Motif5 and Motif7 showed relatively widespread distribution, present in 46 and 27 proteins, respectively. Motif1 was found in only 18 proteins, representing the smallest distribution. From specific observations, 23 CER proteins possessed 1 motif and 4 CER proteins had 2 motifs, among which *LbaCER10-1* contained 18 motifs, while the remaining proteins contained 3-9 motifs. However, 41 CER proteins lacked any motifs, suggesting that the goji *CER* genes may have experienced loss or acquisition of conserved motifs during evolution, potentially indicating the emergence of novel functions in evolutionary process.

In Figure 4, exon/intron structure analysis of the goji *CER* genes revealed that 17 genes contained a single exon, 19 genes contained two exons, and 6 genes contained three exons. *LbaCER10-1* possessed 39 exons, representing the highest number, while the remaining genes contained 1-26 exons. The goji *CER* genes exhibited substantial variation in exon/intron numbers, indicating that these members may possess differentiated biological functions.

### 3.5. Cis-Acting Elements Analysis of Goji CER Gene Family Members

**Figure 5:**
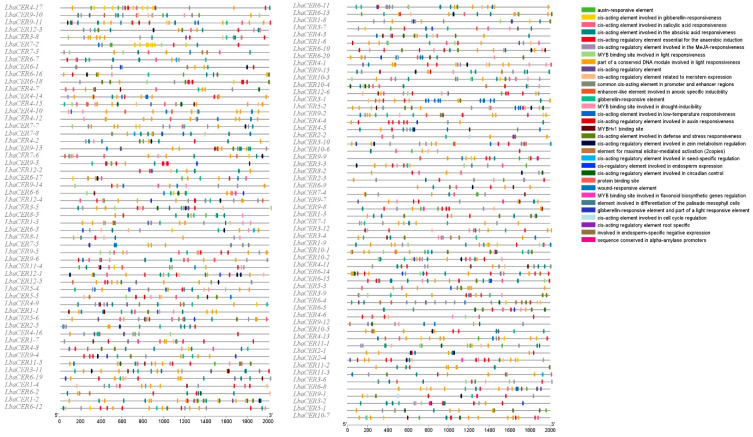
Analysis of cis-acting elements in the goji *CER* gene family members.

As shown in Figure 5 and Supplementary Table S2, the analysis of 2000 bp promoter regions from 113 members of the goji *CER* gene family identified 32 types of cis-acting elements. They mainly included light-responsiveness, MeJA-responsiveness, abscisic acid-responsiveness, and anaerobic induction. Among these, cis-acting elements associated with light-responsiveness, MeJA-responsiveness, and abscisic acid-responsiveness showed relatively high abundance, appearing 353, 316, and 254 times, respectively, accounting for 18.15%, 16.25%, and 13.06% of total occurrences. Notably, root specific appeared only once, representing the lowest abundance. Therefore, the goji *CER* gene family may regulate these elements to enable active responses to various environmental stresses such as strong light, drought, and pest/disease conditions.

### 3.6. Synteny Analysis of Goji CER Gene Family Members

**Figure 6:**
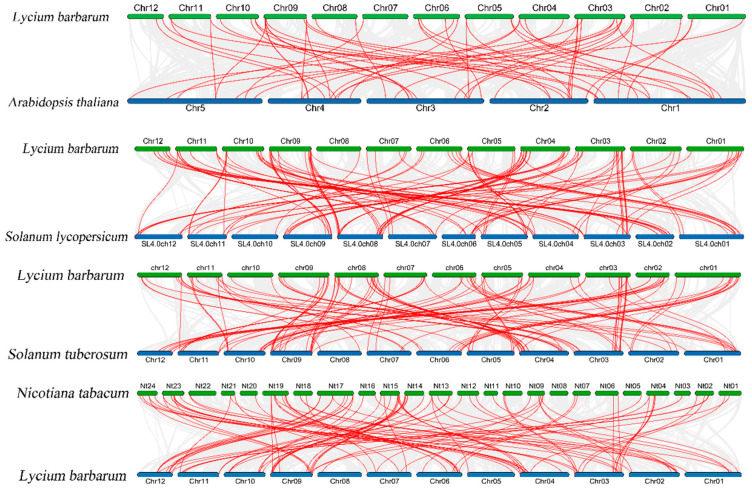
Synteny analysis of goji *CER* gene family members with other species.

To elucidate the evolutionary relationships of the goji *CER* gene family, interspecies synteny analysis was conducted between goji and the model plant *Arabidopsis*, as well as Solanaceae plants including tomato, potato, and tobacco. The results (Figure 6) demonstrated that there were 56 homologous gene pairs between goji and *Arabidopsis*, 110 homologous gene pairs between goji and tomato, 108 homologous gene pairs between goji and potato, and 97 homologous gene pairs between goji and tobacco, indicating that closer phylogenetic relationships correspond to more homologous gene pairs. Statistical analysis of homologous genes among these five species revealed that (Supplementary Table S3) chr3 contained the highest number of homologous gene pairs (54 pairs), while chr9 showed the second highest number (53 pairs), and chr7 contained the fewest homologous gene pairs (18 pairs). Therefore, goji chromosomes 3 and 9 appear to be the most evolutionarily conserved, whereas chromosome 7 exhibits the most significant variation.

### 3.7. Phylogenetic Analysis of Goji *CER* Proteins with Arabidopsis

**Figure 7:**
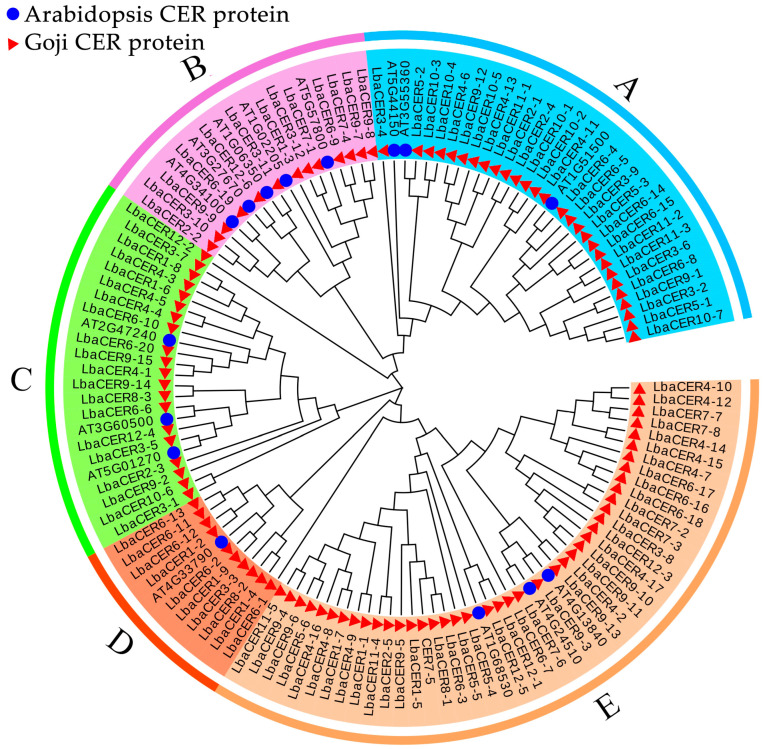
Phylogenetic evolution of the goji CER proteins.

As shown in Figure 7, based on phylogenetic relationships, 15 structurally diverse *Arabidopsis* proteins and 113 goji CER proteins were classified into five subgroups (A–E). Subgroup A consisted of 3 *Arabidopsis* CER proteins (AT1G51500, AT3G55360, and AT5G44150) and 28 goji *CER* gene family member proteins. Subgroup B comprised 5 *Arabidopsis* proteins and 15 goji proteins. Subgroup C contained *Arabidopsis* AT4G33790 and 9 goji proteins. Subgroup D included *Arabidopsis* AT1G68530 and 22 goji proteins. Subgroup E was composed of 5 *Arabidopsis* proteins and 39 goji CER proteins. All goji *CER* gene family members showed phylogenetic relationships with their *Arabidopsis* counterparts, demonstrating the accuracy of the family member selection.

### 3.8. Expression Analysis of Five CER Genes in Different Goji Cultivars and Organs

**Figure 8:**
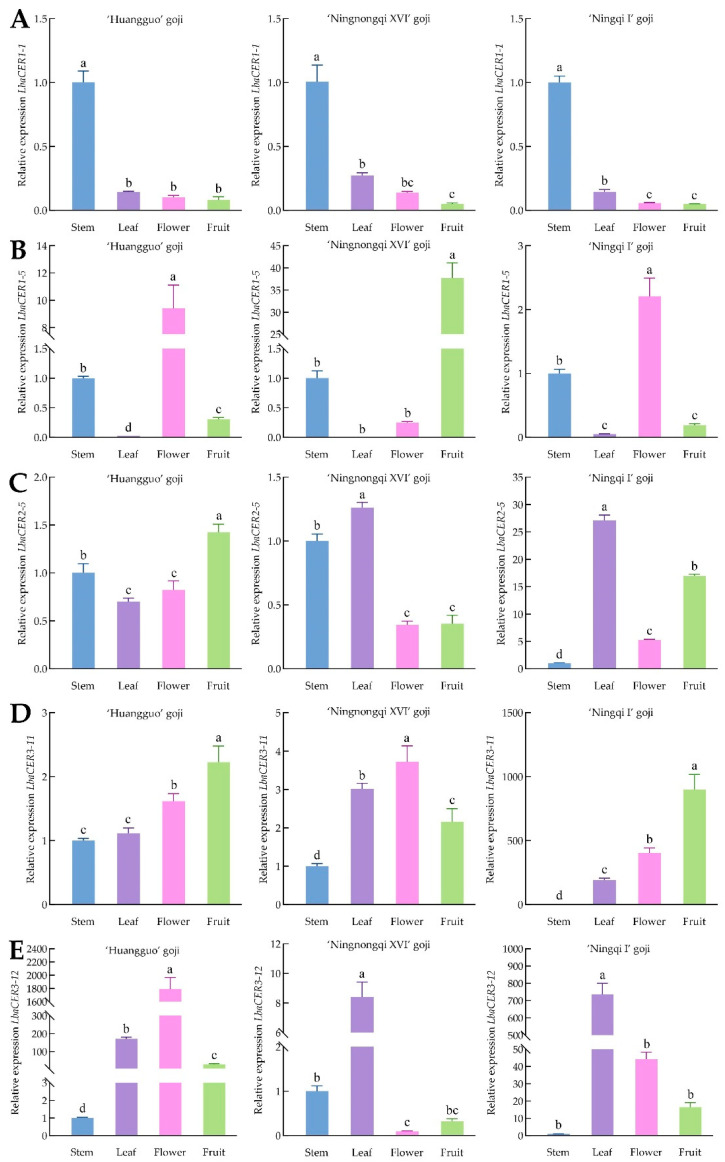
Tissue-specific expression patterns of 5 goji *CER* genes. Note: (**A**) is the relative expression of the *LbaCER1-1* gene; (**B**) is the relative expression of the *LbaCER1-5* gene; (**C**) is the relative expression of the *LbaCER2-5* gene; (**D**) is the relative expression of the *LbaCER3-11* gene; (**E**) is the relative expression of the *LbaCER3-12* gene. The letters above bars represents the differences between data. The same letters indicate non-significant differences, while different letters indicate significant differences.

To further validate the expression patterns of goji *CER* gene family members in different cultivars and organs, five genes with distinct domains were selected for expression analysis at the transcriptional level based on previous transcriptome data, as shown in Figure 8. The *LbaCER1-1* gene exhibited significantly greater expression in stems than in other organs across three cultivars. Both *LbaCER2-5* and *LbaCER3-12* showed significantly greater expression in leaves of ‘Ningqi I’ and ‘Ningnongqi XVI’ compared to other organs. *LbaCER3-11* displayed notably greater expression in flowers of ‘Ningnongqi XVI’, while *LbaCER1-5* was highly expressed in flowers of both ‘Ningqi I’ and ‘Huangguo’ goji. *LbaCER3-12* also showed elevated expression in flowers of ‘Huangguo’ goji. Additionally, both *LbaCER2-5* and *LbaCER3-11* were highly expressed in the fruits of ‘Ningqi I’ and ‘Huangguo’ goji. Combined with results from Figure 1 and Figure 2, we hypothesize that the high expression of *LbaCER1-1* makes it a candidate gene for the greater cuticular wax load on stems of ‘Huangguo’ goji, and the elevated expression of both *LbaCER2-5* and *LbaCER3-12* suggests that they are candidate genes for the greater wax load on leaves of ‘Ningqi I’; additionally, *LbaCER1-1*, *LbaCER2-5*, and *LbaCER3-12* show closer regulatory relationships with cuticular wax deposition in vegetative organs (stems and leaves), and *LbaCER1-5* and *LbaCER3-11* appear more closely associated with wax regulation in reproductive organs (flowers and fruits). Notably, some variations were observed among different cultivars in these expression patterns.

## 4. Discussion

The cuticular wax layer serves as a crucial barrier covering the aerial epidermis of terrestrial plants, protecting them from external environmental stresses [16]. Wax load is often used as an important indicator for screening drought-resistant and pest-resistant germplasms [51]. The production of cuticular wax is regulated by multiple genes, including *CER* [52], *KCS* [53], and *FAR* [54], among others. The *CER* gene family represents key regulators of cuticular wax biosynthesis in plants [55] and has been studied in various species, including apple [56], sunflower [22], jujube [23], passion fruit (*Passiflora edulis*) [57], chestnut [24], cotton (*Gossypium hirsutum*) [55], barley [25], and pepper [26].

The numbers of *CER* gene family exhibit considerable variation among different species [25]. Species with fewer members include apple (10 members) [58], barley (12 members) [25], and cotton (16 members) [55]. Moderately represented species include jujube (29 members) [23], cabbage (*Brassica oleracea*) (32 members) [59], chestnut (34 members) [60], and sunflower (37 members) [22]. Species with abundant members include pepper (79 members) [26]. In the present study, the goji *CER* gene family reached 113 members, substantially exceeding the numbers in the aforementioned species. This discrepancy may be attributed to variations in genome size and various tandem and segmental duplications. In preliminary investigations, we attempted to identify the gene using PF12076 [25] and PF04116 [23] values; however, the results proved unsatisfactory. The PF12076 screening identified only eight members that containing both FA_hydroxylase and Wax2_C domains, while PF04116 failed to identify any relevant members, contradicting current understanding of the *CER* gene family. Consequently, this study employed *Arabidopsis* CER protein structures as templates, initially conducting virtual alignment of similar protein domains, followed by individual manual verification to obtain family members. This technical approach demonstrated accurate and reliable screening of goji *CER* gene family members.

The variations in conserved motifs among gene family members can be used to explain differences in protein functions [60]. Within the same evolutionary clade, *LbaCER* genes exhibited similar motif compositions and exon–intron structures. As shown in Figure 4, LbaCER4-17 through LbaCER9-3 all contained the conserved Motif7, while LbaCER1-5 to LbaCER11-5 shared multiple conserved motifs. Notably, 36 proteins from LbaCER3-11 to LbaCER1-9 showed unique motif losses, potentially related to functional divergence or emergence of novel functions. These motif structural characteristics were largely consistent with *CER* gene families in pepper [26], apple [40], and sunflower [22]. Variations in exon and intron numbers represent an important source of gene family diversity, influencing gene function and expression [61]. Differential exon usage and alternative splicing have been demonstrated to be crucial for functional diversification of *CER* genes [17]. In this study, *LbaCER* genes exhibited substantial variation in intron numbers, ranging from 1 to 39, exceeding the ranges observed in cotton (3–13) [55], sunflower (1–19) [22], and passion fruit (1-28) [57]. Specifically, 17 genes contained a single exon, while *LbaCER10-1* possessed the maximum number of 39 exons. Notably, members with identical or similar motifs and exons clustered well in the phylogenetic tree. These results suggest that LbaCER genes may have undergone multiple rounds of intron loss and gain during evolution, promoting alternative splicing and generating messenger RNAs with diverse functions [62], thereby enhancing functional diversification.

Cis-acting elements serve as crucial factors regulating gene expression levels, providing molecular insights into species evolution and elucidating the interactions between environmental conditions and gene expression [63]. In *Brassica napus* [64] and maize [65], cis-acting elements of *CER* genes have been associated with wax accumulation and drought tolerance signaling pathways. The involvement of ABRE (abscisic acid-responsive element) cis-acting elements in *Arabidopsis* confers enhanced drought stress tolerance [66]. The GARE motif in rice exhibits responsiveness to gibberellins, particularly under conditions of salt and water-deficit stress [67]. The promoters of *CalCER* genes are enriched with hormone-responsive elements and stress-related elements, consistent with their roles in hormone-mediated stress responses [26]. In this study, analysis of cis-acting elements in the goji *CER* gene family identified 353 elements associated with light-responsiveness, 316 elements involved in MeJA-responsiveness, and 254 elements participating in abscisic acid-responsiveness. These findings suggest that goji likely possesses strong tolerance to stresses such as intense light, drought, and pests/diseases.

The relative expression levels of five *CER* gene family members in stems, leaves, flowers, and fruits of the three goji cultivars were detected by qRT–PCR. *LbaCER1-1* exhibited the highest expression in stems across all three cultivars, with stable expression trends (Figure 8). Concurrently, the cuticular wax content in stems of these cultivars was significantly greater than that in other organs (Figure 2), a finding further confirmed by SEM observations (Figure 1). Integrating these results, the *LbaCER1-1* gene likely plays a crucial role in cuticular wax biosynthesis and secretion in goji stems, which may correlate with its drought tolerance characteristics [2]. Combining the relative expression levels of *LbaCER2-5* and *LbaCER3-12* with SEM images and leaf wax load data, these two genes may account for the higher wax accumulation in leaves of ‘Ningnongqi XVI’ and ‘Ningqi I’. The elevated leaf wax load suggests these cultivars may possess enhanced resistance to atmospheric drought [43] and disease susceptibility [44].

Certainly, this study still has some limitations. For instance, the functions of the identified LbaCER genes were all predicted based on computational analysis and have not been functionally validated through gene knockout and/or overexpression approaches. In goji cultivation practice, leaves represent critical organs for water loss and susceptibility to pests and diseases. Higher leaf wax load helps reduce water dissipation and pest attachment. Conversely, fruits are economically valuable parts, and a lower fruit wax load can improve fresh fruit palatability and enhance drying efficiency. Therefore, leaf wax and fruit wax load represent two distinct breeding objectives. In future research, CRISPR/Cas9 technology could be employed to generate *LbaCER* mutants, combined with metabolic network analysis to examine changes in wax content or composition. This would provide a theoretical foundation for improving fresh goji processing and storage, elucidating drought resistance mechanisms, and developing disease-resistant cultivars.

## 5. Conclusions

The wax load on leaf surfaces of ‘Ningqi I’ was significantly greater than that of other cultivars, while the wax load on stem surfaces of ‘Huangguo’ goji was markedly greater than that of both other cultivars and plant organs, which aligned with SEM observations. A total of 113 *CER* gene family members were identified in the goji plants, unevenly distributed across 12 chromosomes, with primary localizations in organelles including the plasma membrane, cytoplasm, chloroplasts, and nucleus, clustering into five subgroups. Among goji CER proteins, Motif5 and Motif7 demonstrated the widest distribution. High-frequency occurrences were observed for cis-acting elements associated with light-responsiveness, MeJA-responsiveness, and abscisic acid-responsiveness. Chr3 and 9 exhibited the highest conservation, while chr7 showed the greatest variation. The elevated expression of *LbaCER1–1* gene potentially explains the higher wax load on ‘Huangguo’ goji stems; similarly, the high expression of *LbaCER2-5* and *LbaCER3-12* genes likely accounts for the increased wax load on ‘Ningqi I’ leaves.

## Figures and Tables

**Table 1:** Primer sequences of five goji *CER* genes and reference gene.

Gene ID	Gene Name	Primer Type	Primer Sequences (5′ to 3′)
Lba01g00543	*LbaCER1–1*	F	CATCACCACGCTAGGACCATTG
		R	TTTCACCTTGGCTTTCAACACTTTC
Lba01g01071	*LbaCER1–5*	F	CCTAGCCCTTCTCTTTCTTCCATTATC
		R	ACAACCCATTCCACTTATCGTATAGC
Lba02g02625	*LbaCER2–5*	F	CTGGTGTGATAGCGGTTGATCTTG
		R	TCTCCGTACTAACAACAACAGCATAC
Lba03g02510	*LbaCER3–11*	F	CAAGTTGAATTGTTATGGACTGGAAGG
		R	GCATTCACAGTTGGTATTGGCATTAC
Lba03g02612	*LbaCER3–12*	F	TCGTGCTTATTATGTGGCTGAAGTC
		R	GTTTGGTTGAGTTTCCCTCTTATGTTG
Loc132636280	*RH37*	F R	GCAGGCAAGTCAGGATTAGCA CGCATAACGAGTCAACCATTCAG

## Data Availability

The goji plants genome data were downloaded from the goji (*Lycium barbarum*) genome database (https://figshare.com/articles/dataset/Goji_genomes_and_the_evolution_of_Lycium_Solanaceae_/20416593, accessed on 6 April 2023). The genome data for the tomato, potato, and tobacco genomes were downloaded from the Sol genomics network (https://solgenomics.net/, accessed on 8 August 2025).

## Supplementary Materials

The following supporting information can be downloaded at https://www.mdpi.com/article/10.3390/genes16111257/s1, Table S1: Information and physicochemical properties of the goji *CER* gene family members; Table S2: Cis-acting elements of the goji *CER* gene family members; Table S3: Synteny analysis of *CER* gene family members in goji berries and other species.

## Abbreviations

The following abbreviations are used in this manuscript:

*CER*	Eceriferum
UV	Ultraviolet Radiation
VLCFAs	Very-long-chain fatty acids
FAR	Fatty acyl reductase
MAH	Medium chain alkane hydroxylase
SEM	Scanning electron microscopy
qRT–PCR	Quantitative real-time polymerase chain reaction
MeJA	Methyl jasmonate
ABA	Abscisic acid
PEG	Polyethylene glycol
VLC	Very long chain
